# The methanolic extract of Cinnamomum zeylanicum bark improves formaldehyde-induced neurotoxicity through reduction of phospho-tau (Thr231), inflammation, and apoptosis

**Published:** 2020-05-25

**Authors:** Sara Sayad-Fathi, Arash Zaminy, Parvin Babaei, Fatemeh Yousefbeyk, Nasibeh Azizi, Ebrahim Nasiri

**Affiliations:** 1 Cellular and Molecular Research Center, School of Medicine, Guilan University of Medical Sciences, Rasht, Iran; 2 Neuroscience Research Center, School of Medicine, Guilan University of Medical Sciences, Rasht, Iran; 3 Department of Physiology, School of Medicine, Guilan University of Medical Sciences, Rasht, Iran; 4 Department of Pharmacognosy, School of Pharmacy, Guilan University of Medical Sciences, Rasht, Iran; 5 Department of Medicinal Chemistry, School of Pharmacy, Guilan University of Medical Sciences, Rasht, Iran

**Keywords:** neurotoxicity, formaldehyde, Cinnamomum zeylanicum, tauopathy, spatial memory

## Abstract

Accumulation of formaldehyde (FA) in the brain is linked to age-related neurodegenerative disorders, as it accelerates memory impairment through tau protein aggregation, inflammation, and nuclear damage. This study aimed to assess the possible effects of methanolic cinnamon extract (CE) on FA-induced neurotoxicity in rats. The animals were treated with CE (100, 200, and 400 mg/kg, P.O.) for 30 days following FA administration (60 mg/kg, I.P.) for 30 days. Briefly, spatial and inhibitory memory were examined by Morris water maze (MWM) and passive avoidance (PA) tasks, respectively. The Nissl, Hoechst, and Bielschowsky silver staining methods were also used to assess apoptosis and neurofibrillary tangles (NFTs) in the hippocampal CA1 region, respectively. Brain tissues were probed with an anti-phospho-tau (Thr^231^) monoclonal antibody to assess tau hyperphosphorylation. Inflammatory cytokines (IL-1β, IL-6, and TNF-α) were also measured by ELISA assay. Western blotting was performed to quantify the amount of phospho-tau (Thr^231^), caspase-8, and caspase-9. The results showed that FA injection significantly caused tau hyperphosphorylation at Thr^231^ residue, which in turn disturbed the MWM performance. The ratio of apoptotic to intact neurons increased following FA treatment. The results of Western blotting indicated that the hippocampal levels of phospho-tau (Thr^231^) and caspase-8 were significantly higher in the FA group compared to the control group. The hippocampal levels of IL-1β, IL-6, and TNF-α in the FA group were also higher than the control group. Administration of 200 mg/kg of CE significantly improved the rats' MWM performance, decreased the levels of phospho-tau (Thr^231^), caspase-8, IL-6, and TNF-α, and reduced the ratio of apoptotic to intact neurons. Overall, cinnamon improved cognitive performance in FA-treated rats by eliminating tau hyperphosphorylation, inflammatory cytokines, and nuclear damage.

## Introduction

Any disturbance in the production, function, or elimination of tau protein is considered a tauopathy. The best example of tauopathy is Alzheimer's disease (AD), associated with abnormal hyperphosphorylation of tau protein (Alzheimer, 1906). Neurofibrillary tangles (NFTs), which mostly consist of phosphorylated tau proteins, are one of the hallmarks of AD, with a significant impact on cognitive impairment (Saha and Sen, 2019[44]). 

The tau protein is a dipole and a mainly cytosolic microtubule-associated protein (MAP), which is expressed in neural cells and provides structural support by promoting microtubule assembly. The tubulin-binding capacity of tau protein is dependent on its phosphorylation state. Under physiological conditions, only a small amount of p-tau (free form) is present in the cytoplasm, whereas in pathological conditions, abnormal phosphorylation of tau protein affects its tubulin-binding capacity and results in microtubule disorganization (Alonso et al., 2018[2]). On the other hand, tau protein is a naturally unfolded protein, which undergoes structural changes during phosphorylation, increasing its potential for self-polymerization and aggregation (Jeong, 2017[18]). Aggregation of phospho-tau results in intracellular NFT formation, which interferes with the axoplasmic flow and synapses. Therefore, tau phosphorylation can be of utmost importance in age-related neurodegenerative disorders.

Formaldehyde (FA) is a well-known pollutant and a natural byproduct of metabolism in both invertebrate and vertebrate cells (Kalapos, 1999[19]). Studies have shown that aging, diet, environmental pollution, and genetic factors can lead to the endogenous accumulation of FA (Tong, 2017[47]). Since this compound can cross the blood-brain barrier, it may play a role in age-related cognitive impairments (Su et al., 2016[46]). It is also a highly reactive agent, which can cause DNA damage by formation of DNA/protein cross-links (Liu et al., 2018[25]) and induction of protein polymerization through reacting with thiol and amino groups. 

A recent study proposed FA as an indicator of aging and cognitive impairment (Tong et al., 2017[49]). It has been also suggested that elevated levels of FA in the brain and cerebrospinal fluid (CSF) can be related to neurodegenerative diseases, such as AD, in human biopsies and animal models (Ono et al., 2005[38]). Abnormally elevated FA leads to cognitive decline through different mechanisms, including tau hyperphosphorylation both *in vitro *and *in vivo* (Lu et al., 2013[28]; Nie et al., 2007[35]). Evidence shows that FA adversely affects the hippocampus (Liu et al., 2010[27]), as one of the most important components of the limbic system in spatial memory (Eichenbaum et al., 1999[12]). Despite major efforts to highlight the role of FA in neurodegeneration, there are few studies on mechanisms suppressing its activity in the brain.

Recently, major attention has been paid to the use of bioactive compounds in herbal medicine for the treatment of different disorders. Plant-based treatments are found to be safer and more cost-effective than chemical drugs and have fewer side effects. *Cinnamomum zeylanicum* (CZ), also known as “true cinnamon” from the Lauraceae family, is one of the two main varieties of the genus *Cinnamomum*. It is a medicinal plant with confirmed effectiveness in the treatment of neurodegenerative disorders. Also, cinnamon is the most commonly used spice throughout the world. According to previous studies, a cinnamon concentration below 0.5 g/kg is safe if administered orally to Wistar rats (Ahmad et al., 2015[1]). On the other hand, concentrations of 100, 200, and 400 mg/kg in rats (equivalent to 600-2400 mg/kg in adult humans) do not cause significant neurological changes (Anand et al., 2010[5]). 

Moreover, different parts of cinnamon contain different chemical constituents. It has been shown that the stem bark of CZ contains 60-75 % cinnamaldehyde (CA) (WHO, 1999[51]). In a previous study, CA exhibited the most anti-inflammatory activity in CZ, followed by 2-methoxycinnamaldehyde, α-methyl cinnamaldehyde, eugenol, and cinnamyl alcohol (Ho et al., 2013[16]). *In vivo*, CA is metabolized into cinnamic acid due to oxidation in the liver and is then converted to benzoate in form of sodium salt or benzoyl-CoA by further β-oxidation (Khasnavis et al., 2012[20]). 

CA (1.25 mg/kg, daily) has been approved as a safe natural compound by the Food and Drug Administration (FDA) and the Council of Europe (Zhu et al., 2017[53]). It has been proposed that cinnamon compounds can cross the blood-brain barrier with respect to the extent of lipophilicity (Frydman-Marom et al., 2011[13]; Lin et al., 2007[23]). Catechin, a polyphenol in cinnamon and grape seed, as well as its metabolites, has been also identified in brain tissues after acute and chronic oral administration (Wang et al., 2012[50]). The free radical scavenging activity of cinnamon polyphenol derivatives has been also reported in the literature (Roussel et al., 2009[43]; Niknezhad et al., 2019[36]). 

Studies have shown that CZ increases neuronal survival by rebalancing the redox state and suppressing neuroinflammation. Therefore, targeting the hallmarks of AD can improve both histopathological and behavioral outcomes in different animal models (Momtaz et al., 2018[33]; Madhavadas et al., 2017[29]; Modi et al., 2015[31]; George et al., 2013[14]; Jana et al., 2013[17]; Frydman-Marom et al., 2011[13]) and *in vitro* (Peterson et al., 2009[39]). With this background in mind, the present study aimed to investigate the effect of methanolic extract of CZ bark on the hippocampal phospho-tau, inflammatory cytokines, nuclear damage, and spatial memory of FA-treated rats.

## Methods

### Cinnamon extract preparation

The dried CZ bark was purchased from a local store. After authentication by the Department of Pharmacognosy (Faculty of Pharmacy, Guilan University of Medical Sciences, Rasht, Iran), the bark (Herbarium No: GUMS-C17) was grounded into a moderately coarse powder, and an alcoholic extract was obtained by percolation at 35 °C with methanol (Carlo Erba, Italy) three times for 24, 48, and 72 hours, respectively. The extract was then filtered through a Whatman filter paper (GE Healthcare, UK) and concentrated using a rotary evaporator (Heidolph Instruments, Germany). Finally, the concentrated extract was freeze-dried (Martin Christ GmbH, Germany), and the dark brown lyophilized powder was stored at -20 °C.

### Phytochemical analyses

#### Total phenolic content assay

The total phenolic content of the extract was determined using the Folin-Ciocalteu method, with gallic acid (GA) as a standard (Ghasemi et al., 2018[15]). Briefly, 1 mL of the extract (1 mg/mL) was incubated with 5 mL of the Folin-Ciocalteu reagent (10-fold dilution) for 10 minutes at room temperature. Then, it was mixed with 4 mL of sodium bicarbonate solution (75 g/L) for 30 minutes at room temperature, and absorbance was read at 765 nm, using a UV/VIS spectrophotometer (Lambda 25, Perkin Elmer, USA). The total phenolic content was calculated according to the standard calibration curve of GA, obtained by measuring the absorbance of five known concentrations of the GA standard (25, 50, 70, 100, and 200 µg/L). Data are presented as μg gallic acid equivalent (GAE) per mg of dry extract. All samples and readings were prepared and measured in duplicate. 

#### High-performance liquid chromatography (HPLC)

The HPLC analysis was performed using a Smartline HPLC apparatus (Knauer, Germany), equipped with a UV detector, a reversed-phase C18 column (S5 ODS2, 4×250 mm, 5 µm; Waters Spherisorb, USA), and a Security Guard column (S5 ODS2, 4.6×10 mm; Waters Spherisorb, USA). The mobile phase consisted of a linear gradient of 0.1 % phosphoric acid in water (solvent A) and acetonitrile (solvent B). The eluted ratio was adjusted with 10 % acetonitrile, and then, 20 % acetonitrile within 12 minutes. Next, it was replaced with 50 % acetonitrile within 23 minutes (35 minutes in total) and with 100 % acetonitrile within five minutes (40 minutes in total). Chromatography continued with 100 % acetonitrile for another five minutes. The flow rate of the mobile phase was measured to be 1.0 ml/min. Also, the injection volume of the samples and standard solutions was 10 μL. The UV/VIS spectra were recorded at 265 nm (Ding et al., 2011[11]).

To quantify the amount of cinnamaldehyde, trans-CA (Merck, Germany) was used as an external standard. A calibration curve was plotted using eight different concentrations of trans-CA (0.25, 0.5, 1, 2.5, 5, 10, 15, and 20 mg/mL), and each concentration was measured in triplicate. The peak areas of the samples were correlated with the concentrations, according to the standard calibration curve.

### Animals and treatments

In this original study, male Wistar rats (6-8 weeks old; 200-230 g) were purchased from Pasteur Institute of Iran. The animals were housed in a temperature-controlled room (25 °C) with a 12-hour photoperiod and *ad libitum* access to food and water. After two weeks of acclimatization, they were randomly assigned to six groups (n=7 per group). The sham group received no intervention, whereas the FA group received 60 mg/kg of FA (I.P.) for 30 days. Also, the three experimental groups received 100 (CE_100_), 200 (CE_200_), and 400 (CE_400_) mg/kg of the extract by syringe feeding (Atcha et al., 2010[6]) one hour after FA treatment. On the other hand, the control group received the cinnamon extract solvent (10 % DMSO/10 % sucrose) via syringe feeding after FA treatment. 

After behavioral assessment, the animals were decapitated under deep anesthesia with a ketamine/xylazine cocktail, and the brain tissues were removed. Samples for histological analysis were blocked in paraffin following fixation in 10 % neutral buffered formalin. Also, for Western blotting and ELISA assay, the dissected hippocampi were stored at -80 °C after snap freezing in liquid nitrogen.

### Behavioral assessments

#### Morris water maze (MWM) task

The MWM task is used to assess spatial memory (Morris, 1984[34]). A circular black pool (diameter=184 cm), filled half way with water (20-22 °C), was used for this task. A hidden platform was submerged 2-3 cm below the water surface in a fixed position in the middle of the target quadrant. The animals were exposed to four acquisition trials per day for four consecutive days. They were released around the pool at different starting points while facing the wall to navigate a path to the hidden platform within 90 seconds or less. This trial was followed by a probe trial on day five, when the animals were allowed to search the pool for the removed hidden platform at a starting point positioned at a 180° angle opposite to the target quadrant for 90 seconds. All procedures were tracked using a camera, connected to EthoVision software (Noldus 7.1, Netherlands), which allowed us to analyze the escape latency, the spent time in the target quadrant, and the swimming speed. 

#### Passive avoidance (PA) task

The PA task was performed using a shuttle box to assess avoidance memory consolidation (Amirshabani et al., 2017[4]). The shuttle box, which was a rectangular chamber (20×20×30 cm), consisted of two light and dark compartments with grid floors, enabling the delivery of electric shock; the compartments were separated by a door. In the acquisition trial, the animals were placed in the light compartment and allowed to explore it for 20 seconds; next, the door was lifted. After the animal entered the dark compartment with all four paws, the door was closed, and a foot shock (1 mA, 50 Hz, 5 sec) was delivered through the grid floor. The animals were then returned to their home cages. The retention trial was performed after 24 hours. In this trial, the animals were placed in the light compartment with the door open and allowed to explore it for 180 seconds. Finally, the step-through latency and the spent time in the dark compartment were recorded.

### Histological analysis

Histological sections (7 μm) were obtained using a rotary microtome (microTEC, Germany). The temperature-modified Bielschowsky silver staining method was used to assess the possible formation of NFTs. The number of intact neurons was assessed via Nissl staining, while apoptosis was assessed via Hoechst staining. Also, the phosphorylation state of tau protein was examined by immunohistochemistry (IHC), using a monoclonal antibody against the Phospho-Tau (Thr^231^) Antibody (Abcam, UK). All histological analyses were performed using light/fluorescence microscopy (Olympus, Japan).

#### Temperature-modified Bielschowsky silver staining

This staining method was used to assess the possible formation of NFTs (Litchfield and Nagy, 2001[24]). All jars were washed with nitric acid and deionized water, respectively. The brain sections were deparaffinized, rehydrated, and incubated with 20 % silver nitrate (AgNO_3_) for 20 minutes in darkness at 5 °C. After washing in deionized water, the sections were incubated with an ammoniacal silver nitrate solution for another 20 minutes in darkness at 5 °C. Ammonia was added drop by drop until dark brown precipitates appeared and then vanished. The sections were then rinsed with ammonia water (200 μl/100 mL). 

The developing solution (0.5 g of citric acid, 20 mL of formaldehyde, 50 μL of concentrated HNO_3_, and 100 mL of distilled water) was added to the previously prepared ammoniacal silver nitrate solution (50 µl/25 mL), and the sections were incubated in it for five minutes. After immersion in ammonia water, the sections were washed in deionized water (x2), and 5 % sodium thiosulfate was used to terminate the reaction. The slides were then dehydrated, cleared, and mounted with a permanent mounting medium (Millipore, Germany). To estimate NFTs in the CA1 region of the hippocampus, the stained sections were assessed in five random visual fields (400x magnification).

#### Nissl staining 

This method was applied to assess the number of intact neurons. The brain sections were deparaffinized, rehydrated, and stained with 0.5 % aqueous Cresyl Violet solution for five minutes. The slides were then dehydrated, cleared, and mounted with a permanent mounting medium. To quantify the number of intact neurons in the CA1 region of the hippocampus, the number of pyramidal neurons was counted by two researchers in five random visual fields (400x magnification). Data are presented as the mean (±SD) number of intact neurons per group.

#### Hoechst staining 

To estimate the nuclear damage, Hoechst 33342 stain was used. The brain sections were deparaffinized, rehydrated, and stained, according to the manufacturer's instructions (Invitrogen, USA). The slides were then mounted using a mounting medium (90 % glycerol, 20 mM Tris, pH=8.0). To estimate the ratio of apoptotic neurons (with condensed/fragmented nuclei) in the CA1 region of the hippocampus, the number of pyramidal neurons, with and without nuclear damage, was counted by two researchers in five random visual fields (400x magnification). Data are presented as the ratio of the mean (±SD) number of apoptotic neurons to intact neurons in each group. 

#### IHC analysis

The IHC analysis was performed to assess the phosphorylation state of tau proteins. The paraffin-embedded tissue (PET) blot method was used as an alternative method for antigen retrieval, where PET sections were collected on a polyvinylidene fluoride (PVDF) membrane (Millipore, Germany) (Moh et al., 2010[32]). After drying in a 60 °C oven for 30 minutes and immersion in two changes of xylene, absolute ethanol, 95 % ethanol, 70 % ethanol, and Tris-buffered saline (TBS; 150 mM NaCl, 50 mM Tris, pH=7.6) for five minutes, the PET blots were incubated with 125 μL of hydrogen peroxide 3 % at room temperature to remove endogenous peroxidases. 

After rinsing in TBS, the PET blots were blocked with 10 % normal goat serum (NGS) for 30 minutes. Next, an Anti-Phospho-Tau (Thr^231^) Primary Antibody (1:200) was used, and the PET blots were allowed to incubate overnight at room temperature. After one day, the PET blots were rinsed for one minute in a large volume of TBS, and then, HRP-conjugated goat anti-rabbit secondary antibody (1:500; Abcam, UK) was applied to the blots. The stain was developed using a 3,3'-diaminobenzidine (DAB) kit (Abcam, UK). Finally, the PET blots were incubated with a mixture of dimethyl sulfoxide (DMSO) and ethanol (3:2) for three minutes to achieve transparency for evaluation under a light microscope. To estimate the tau protein hyperphosphorylation in the CA1 region of the hippocampus, the stained sections were assessed in five random visual fields (400x magnification).

### Evaluation of inflammatory cytokines

The hippocampal concentrations of interleukin-1β (IL-1β), IL-6, and tumor necrosis factor-α (TNF-α) were estimated, using commercially available ELISA kits (Aviva Systems Biology, USA), according to the manufacturer's instructions.

### Western blotting 

The hippocampi (n=3 per group) were homogenized in lysis buffer, containing protease and phosphatase inhibitor cocktails. The protein content was determined as described by Bradford (1976[8]). Equal amounts (60 μg) of the samples or the pre-stained protein ladder (Thermo Fisher Scientific, USA) were electrophoretically separated on SDS-polyacrylamide gel and transferred to the PVDF membranes. The blots were then blocked with 2 % skimmed milk, which was dissolved in TBS containing 0.1 % Tween 80 (TBST) for 75 minutes and probed overnight with an Anti-Phospho-Tau (Thr^231^) Primary Antibody (1:1000) at 4 °C while shaking. 

After washing three times with TBST, the blots were incubated with HRP-conjugated goat anti-rabbit secondary antibody (1:15000) for 75 minutes at room temperature on a shaker, followed by washing in TBST in triplicate. The blots were stripped and reprobed with polyclonal antibodies (1:1000) against caspase-8 (Abcam, UK), caspase-9 (Abcam, UK), and β-actin (RatzFatz, Germany). Afterward, the immunoreactive protein bands, detectable by enhanced chemiluminescence (ECL) reagents (BioRad, USA), were exposed to radiographic films. Finally, the autoradiograms were scanned, and densitometry was performed using ImageJ software.

### Statistical analysis 

Distribution of data was analyzed by Shapiro-Wilk test. The MWM results were analyzed using one-way ANOVA and repeated measures ANOVA, followed by Tukey's post-hoc test. All other quantitative variables were analyzed using one-way ANOVA and Tukey's post-hoc test. Differences were considered statistically significant at P<0.05. Data analyses were performed in GraphPad Prism Version 6.

### Ethical considerations

All experiments were performed in accordance with the guidelines of Guilan University of Medical Sciences for experimental ethics and handling of laboratory animals (approval code: 94041617; ethics code: IR.GUMS.REC.1394.187), which are in line with the National Institutes of Health (NIH) Guide for the Care and Use of Laboratory Animals (NRC, 2011[37]).

## Results

### Phytochemical analyses

#### Total phenolic content assay

The total phenolic content of CZ extract was calculated using a linear equation, based on the standard curve of GA (y=0.0088x - 0.0388; R^2^=0.997) and reported as 68.02±0.0036 mg GAE/g of dry extract.

#### HPLC

**Figure 1:**
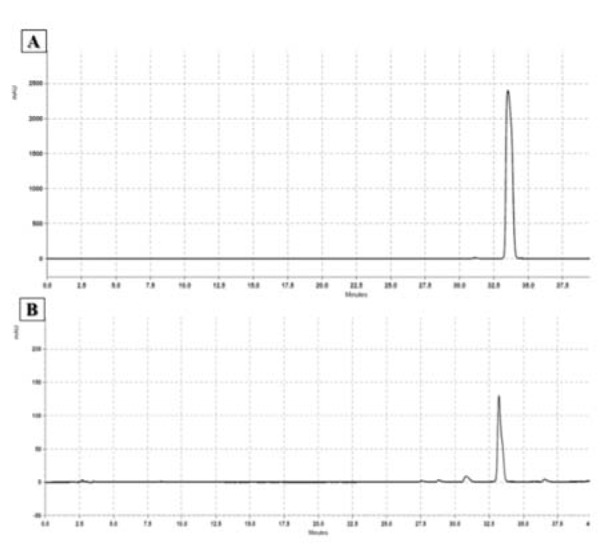
Result of HPLC using trans-cinnamaldehyde as internal control. The mobile phase consisted of a linear gradient of 0.1 % phosphoric acid in water and acetonitrile. The flow rate of the mobile phase was 1.0 ml/min. The injection volume of standard (A) and sample solutions (B) was 10 μL. The UV/Vis spectra were recorded at 265 nm. The peak areas of the samples were correlated with the concentrations according to the standard calibration curve.

The CA content of the CZ extract was quantified by HPLC (Figure 1(Fig. 1)). The calibration curve (y=3E-0.7x - 0.4673) was plotted, and excellent linearity was obtained for the trans-CA analytical standard between the peak areas and concentrations of 0.25-20 mg/mL (R^2^=0.9994). The results showed that the methanol extract contained 82.66 mg of CA per gram of dry extract.

### Behavioral assessments

#### MWM task

**Figure 2:**
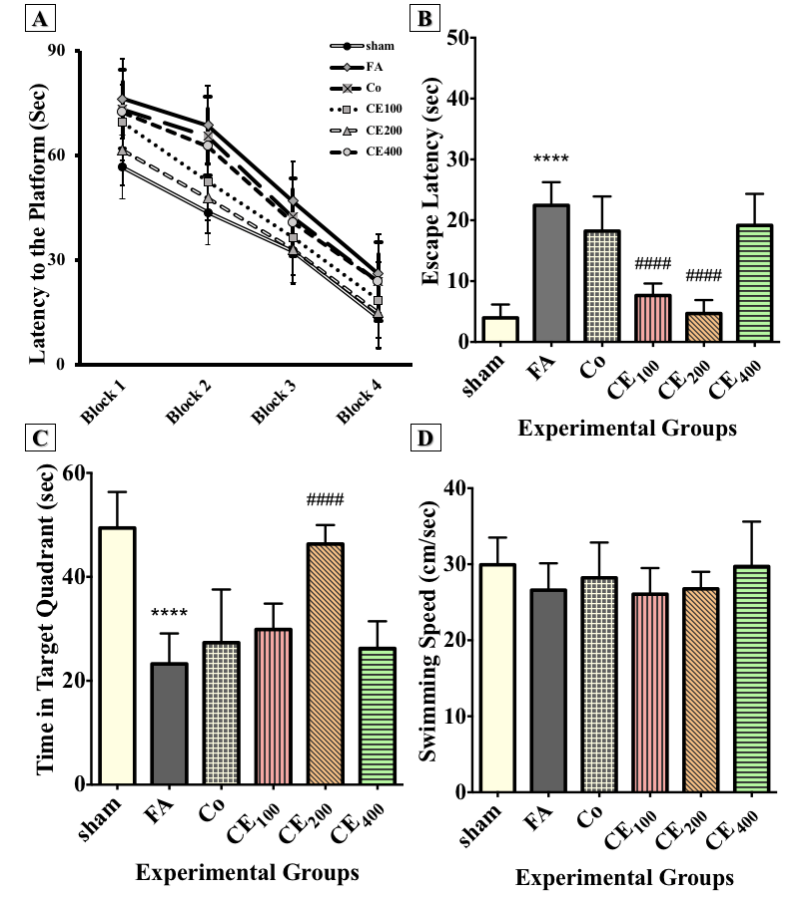
Effect of CE administration on spatial memory formation in the Morris water maze task. All animals learned to swim toward the hidden platform during acquisition trials (A). In the probe trial, the FA group showed a significant delay in finding the hidden platform and spent noticeably less of their time in the target quadrant (B, C). Co-treatment with CE at doses of 100 and 200 mg/kg decreased the escape latency, but the time spent in the target quadrant increased significantly only in the group treated with 200 mg/kg of CE. There were no significant changes in the swimming speed of animals among groups (D). ^****^p<0.0001 when compared to control, ^####^p<0.0001 when compared to FA sham: no treatment, FA: formaldehyde 60 mg/kg, Co: FA 60 mg/kg + solvent, CE_100,200,400_: FA 60 mg/kg + methanolic cinnamon extract 100, 200, 400 mg/kg

The results showed that all animals learned to use spatial clues to find the path to the hidden platform during the acquisition trials (Figure 2A(Fig. 2)). In the probe trial, the FA group showed a significant delay in finding the hidden platform (P<0.0001) (Figure 2B(Fig. 2)) and spent a noticeably less amount of time in the target quadrant (P<0.0001) (Figure 2C(Fig. 2)). The CE co-treatment at concentrations of 100 and 200 mg/kg decreased the escape latency (P<0.0001 for both concentrations). The time spent in the target quadrant improved only in the group treated with 200 mg/kg of CE (P<0.0001). However, there was no significant change in the swimming speed of animals in the groups (Figure 2D(Fig. 2)).

#### PA task

**Figure 3:**
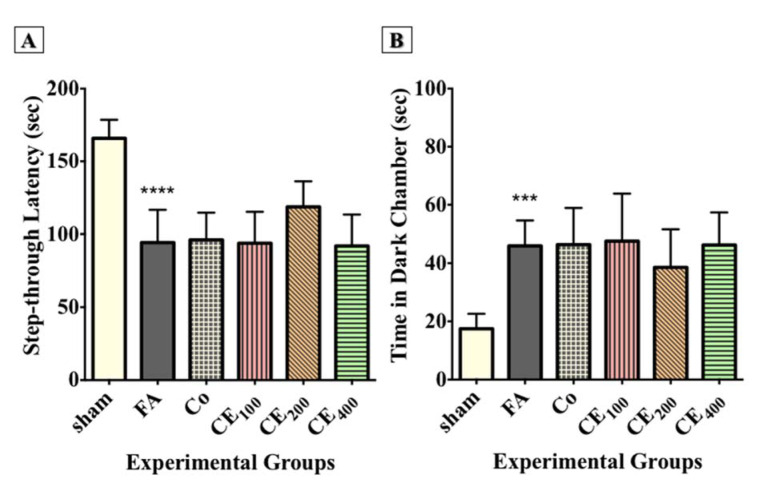
Effect of CE administration on inhibitory memory consolidation in the passive avoidance task. FA administration significantly affected the step-through latency (A) and time spent in the dark chamber (B). Although co-treatment of CE at the dose of 200 mg/kg relatively improved the step-through latency and time spent in the dark chamber, the changes were not statistically significant. ^***^p<0.001, and ^****^p<0.0001 when compared to control sham: no treatment, FA: formaldehyde 60 mg/kg, Co: FA 60 mg/kg + solvent, CE_100,200,400_: FA 60 mg/kg + methanolic cinnamon extract 100, 200, 400 mg/kg

The results showed that FA administration significantly affected the step-through latency (Figure 3A(Fig. 3)) and the time spent in the dark compartment (P<0.0001 and P<0.001, respectively) (Figure 3B(Fig. 3)). Although co-treatment with 200 mg/kg of CE relatively improved the step-through latency and the time spent in the dark compartment, the changes were not significant (Figure 2D(Fig. 2)).

#### Nissl staining 

**Figure 4:**
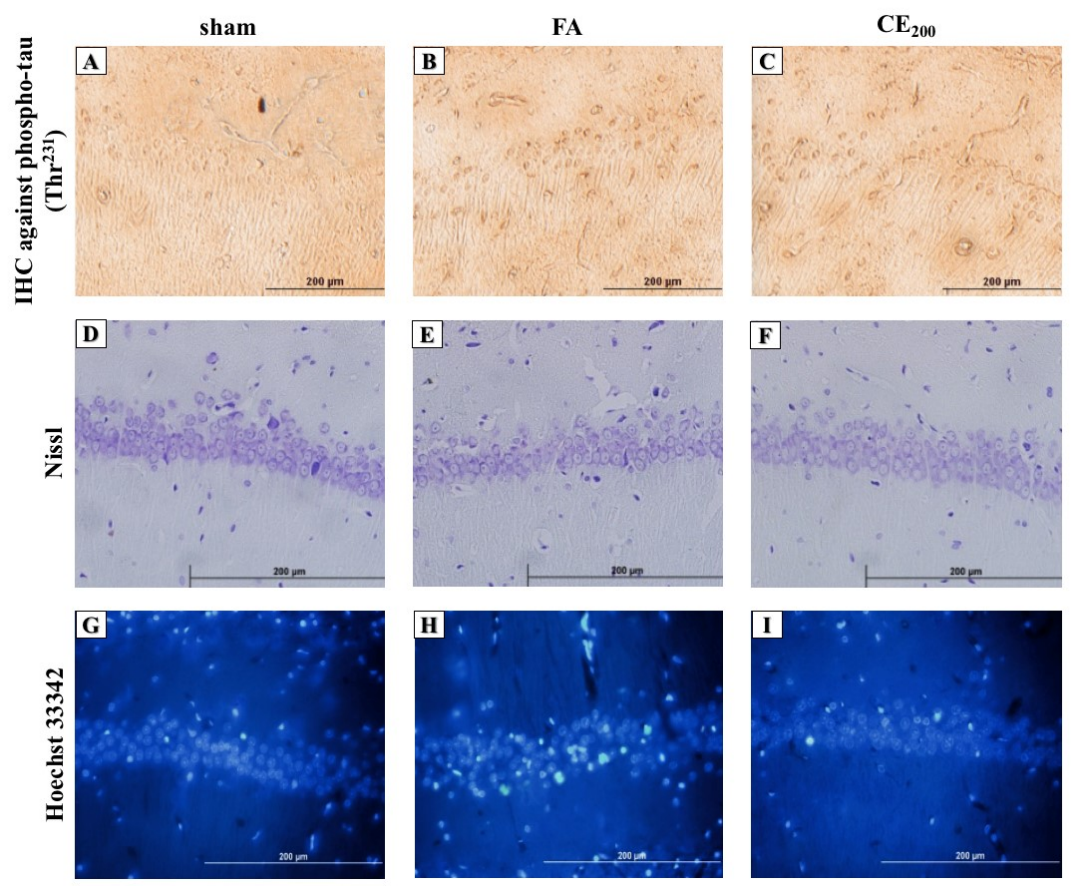
Histological alterations of the CA_1_ area of the hippocampus following treatments (200x). Formaldehyde administration caused a prominent increase in the amounts of phospho-tau in the cytoplasm of the pyramidal cells in the CA_1_ area of the hippocampus (B) compared to control (A). Cells representing nuclear damage are shown to be increased in the FA group (H) compared to control (G). When treated with CE at the dose of 200 mg/kg the amounts of phospho-tau (C) and cells with nuclear damage (I) notably decreased. There were no differences in cell morphology between groups (D, E, F). sham: no treatment, FA: formaldehyde 60 mg/kg, CE_200_: FA 60 mg/kg + methanolic cinnamon extract 200 mg/kg.

**Figure 5:**
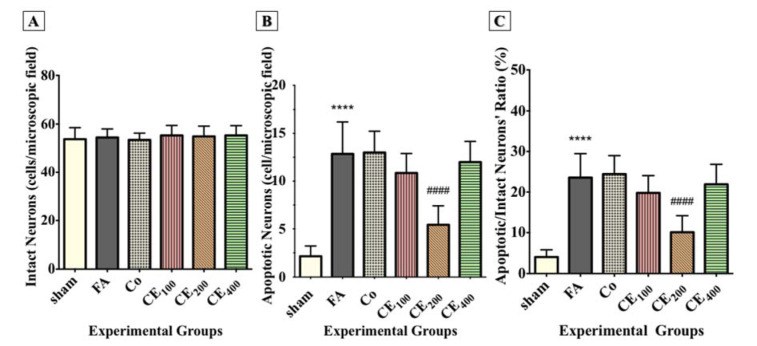
Changes in the numbers of intact neurons, apoptotic neurons, and the ratio of apoptotic/intact neurons in the CA_1_ area of the hippocampus following treatments. Formaldehyde administration caused a significant increase in the numbers of cells representing nuclear damage (implying apoptosis) (B) and the ratio of the apoptotic/intact neurons (C) in the CA_1_ area of the hippocampus. There were no significant differences in the numbers of intact neurons between groups (A). ^****^p<0.0001 when compared to control. ^####^p<0.0001 when compared to FA sham: no treatment, FA: formaldehyde 60 mg/kg, Co: FA 60 mg/kg + solvent, CE_100,200,400_: FA 60 mg/kg + methanolic cinnamon extract 100, 200, 400 mg/kg.

Although the number of intact neurons in the CA1 region did not decrease due to FA administration, there was no significant difference between the groups (Figure 4D, 4E, 4F(Fig. 4), and Figure 5A(Fig. 5)).

#### Hoechst staining 

Following FA administration, there was a significant increase in the number of apoptotic neurons and their ratio to intact neurons (P<0.0001 for both) (Figure 4G, 4H, 4I(Fig. 4), Figure 5B, and 5C(Fig. 5)). Almost all three concentrations of CE could eliminate apoptosis, but only the concentration of 200 mg/kg was statistically significant (P<0.0001).

#### IHC analysis

The FA treatment caused a noticeable increase in the color density of pyramidal cell cytoplasm in comparison with the control group, and co-treatment with 200 mg/kg of CE ameliorated the FA-induced tau hyperphosphorylation at Thr^231^ (Figure 4A, 4B, and 4C(Fig. 4)).

### Histological analyses

#### Temperature-modified Bielschowsky silver staining

The results showed no significant differences between the groups (data not shown).

### Evaluation of inflammatory cytokines 

**Figure 6:**
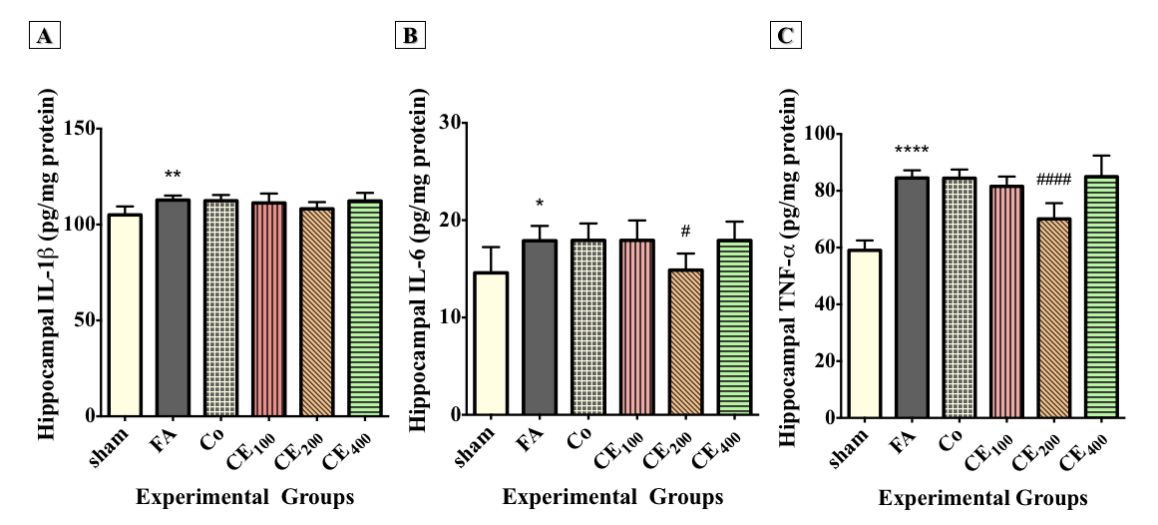
Changes in the hippocampal levels of inflammatory cytokines among groups. Formaldehyde treatment significantly increased the hippocampal levels of IL-1β (A), IL-6 (B), and TNF-α (C) compared to control. Administration of CE at the dose of 200 mg/kg significantly eliminated the hippocampal levels of IL-6 and TNF-α. Slight and insignificant decreases in the levels of IL-1β were also seen. ^*^p<0.05, ^**^p<0.01, and ^***^p<0.001 when compared to control, ^#^p<0.05 and ^####^p<0.0001 when compared to FA sham: no treatment, FA: formaldehyde 60 mg/kg, Co: FA 60 mg/kg + solvent, CE_100,200,400_: FA 60 mg/kg + methanolic cinnamon extract 100, 200, 400 mg/kg.

The hippocampal levels of IL-1β (Figure 6A(Fig. 6)), IL-6 (Figure 6B(Fig. 6)), and TNF-α (Figure 6C(Fig. 6)) significantly increased following FA treatment (P<0.01, P<0.05, and P<0.0001, respectively). Although treatment with 200 mg/kg of CE decreased the hippocampal level of inflammatory cytokines, the reduction of only IL-6 (P<0.05) and TNF-α (P<0.0001) was significant. There was also a slight change in the hippocampal level of IL-1β in response to CE co-treatment, but it was not statistically significant.

### Western blotting 

**Figure 7:**
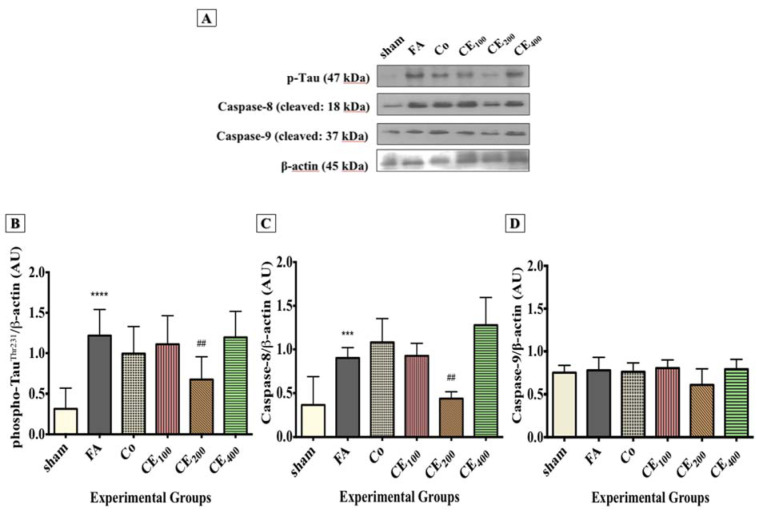
Changes in the hippocampal levels of phospho-tau and apoptosis markers among groups. Levels of phospho-tau (A, B) and Caspase-8 (A, C) were found to be significantly higher in the FA group. Although Caspase-9 (A, D) levels were also increased, the changes were not statistically significant. Administration of CE at the dose of 200 mg/kg significantly eliminated the hippocampal levels of phospho-tau and Caspase-8. Slight and insignificant decreases in the levels of Caspase-9 were also seen. ^***^p<0.001, and ^****^p<0.0001 when compared to control, ^##^p<0.01 when compared to FA sham: no treatment, FA: formaldehyde 60 mg/kg, Co: FA 60 mg/kg + solvent, CE_100,200,400_: FA 60 mg/kg + methanolic cinnamon extract 100, 200, 400 mg/kg.

In line with the IHC results, Western blot analysis also showed that tau phosphorylation significantly increased in response to FA treatment (P<0.0001), and CE co-administration at 200 mg/kg significantly prevented the FA-induced tau hyperphosphorylation (P<0.01) (Figure 7A and 7B(Fig. 7)). The level of cleaved caspase-8 increased in response to FA treatment (P<0.001), whereas treatment with 200 mg/kg of CE significantly eliminated it (Figure 7A and 7C(Fig. 7)) (P<0.01). There was no significant difference in the hippocampal level of caspase-9 between the experimental groups. 

## Discussion

Recent studies have proposed endogenous FA as an indicator of cognitive performance. Researchers believe that FA plays a pivotal role in age-related neurodegenerative disorders, such as AD, and may be an influential factor in deterioration of the patient's condition. Different mechanisms through which FA adversely affects memory have been investigated, including tau hyperphosphorylation. It has been shown that FA interacts with tau protein both directly and indirectly, leading to protein self-polymerization and aggregation both *in vitro* and *in vivo* (Lu et al., 2013[28]; Nie et al., 2007[35]). 

The tau protein is a naturally unfolded MAP protein, which provides structural support for cells through binding to microtubules. However, it loses its capacity to bind to microtubules following phosphorylation, and consequently, the amount of free cytoplasmic phospho-tau increases (Mandelkow et al., 1995[30]). Also, the increment in cytoplasmic phospho-tau increases the possibility of protein self-polymerization and aggregation. Finally, NFTs form and compromise the intracellular transportation. 

Currently, researchers have focused on approaches targeting the primary pathogenic processes before the development of any signs. Lifestyle modifications, such as increasing the level of physical activity and adhering to a healthy diet, are among the most successful approaches. Cinnamon, with significant anti-inflammatory and antioxidant activities, is attracting more and more attention due to its widespread use as a flavoring agent worldwide. In the present study, the effects of the methanolic extract of CZ on histopathological and biochemical changes of the hippocampus and memory were investigated in FA-treated rats. The results indicated that a series of histopathological, biochemical, and cognitive changes occurred in response to FA treatment. It was also observed that the amount of hippocampal phospho-tau, IL-1β, IL-6, and TNF-α significantly increased in the FA group. These changes could explain why spatial memory formation and avoidance memory consolidation were disturbed. 

The review of the literature confirmed the prominent role of increased endogenous FA in tau phosphorylation, neuro-inflammation, and memory deficits in the AD-like brain of rats. In a recent study, Liu et al*.* (2018[26]) showed that FA-exposed C57BL/6 mice developed more phospho-tau in the cerebral cortex, compared to the controls. The MWM results showed a significant delay in the escape latency, and the mice spent a significantly less amount of time in the target quadrant. The brain levels of IL-1β and TNF-α increased in response to FA treatment, indicating inflammation as the underlying mechanism of tau hyperphosphorylation and spatial memory impairment. 

Moreover, Tong et al. (2013[48]) reported spatial memory impairment due to the suppression of long-term potentiation in the hippocampus following chronic FA injection over 30 days. The detected mechanism mimicked age-related memory impairment, caused by the accumulation of endogenous FA in the brain. Moreover, Yang et al. (2014[52]) have shown that methanol feeding for six weeks resulted in impaired spatial memory and tau hyperphosphorylation in the hippocampi of mice. By performing an additional *in vitro* experiment, they confirmed the role of FA, but not other methanol metabolites, such as acetaldehyde or formic acid. Avoidance memory consolidation was also adversely affected by FA treatment. 

In the current study, despite no visible changes in the morphology and number of intact neurons in the CA_1_ region, the ratio of apoptotic neurons (cells with chromatin condensation indicative of nuclear damage) to intact neurons significantly increased following FA administration. Although the hippocampal level of cleaved caspase-9 showed a small insignificant increase, the Western blot analysis indicated that the expression of cleaved caspase-8 was significantly higher in the FA group; therefore, the extrinsic pathway of apoptosis was activated. In line with our study, Sayyar et al. (2018[45]) showed that FA administration increased apoptosis in the CA_1_, CA_2_, and dentate gyrus regions of the rat hippocampus. 

In previous studies, it was found that FA induced apoptosis in the hippocampus, and 10 % of neurons contained condensed chromatins (Yang et al., 2014[52]). Although some researchers have studied the effects of FA treatment on the expression of apoptosis-related proteins, such as Bax, Bcl-2, caspase-3, and caspase-9, to the best of our knowledge, the present study is the first to report the upregulation of caspase-8 following FA treatment. We believe that FA triggers the extrinsic pathway of apoptosis and decreases neuronal survival in the brain.

The methanolic CZ extract used in the present study contained 68.02±0.0036 μg phenolic content per mg of dry extract. The animals in the experimental groups received 1700 μg (100 mg/kg) to 6800 μg (400 mg/kg) of polyphenols, including catechins, according to their body weight every day for 30 days. The HPLC results also indicated that the amount of CA, which is responsible for most anti-inflammatory properties and flavor of cinnamon (Rao and Gan, 2014)[42], was 82.66 mg/g dry extract. In previous studies, the median lethal dose (LD_50_) of CA was 1850±37 mg/kg in Wistar rats, which is comparable with 11.4±0.2 g/kg in adult humans (Ranasinghe et al., 2012[41]; Babu et al., 2007[7]). 

In this regard, Kim et al. reported that CA at doses of 2 and 6 mg/kg significantly inhibited the activation of NF-κB in rats through NIK/IKK, ERK, and p38 MAPK pathways (Kim et al., 2007[21]). Trans-CA has also shown inhibitory effects on neuroinflammation by downregulating iNOS, COX-2, and TNF-α other than NF-κB (Pyo et al., 2013[40]; Chen et al., 2016[10]). Our results showed that 200 mg/kg of CE significantly improved the impact of FA on the MWM indices. Although administration of CE at a concentration of 100 mg/kg significantly decreased the escape latency, the time spent in the target quadrant was not significantly different between this group and the FA group. 

At the molecular level, our results suggested that 200 mg/kg of CE significantly reduced the elevated hippocampal levels of IL-6 and TNF-α in the FA group. However, the level of IL-1β, which increased in response to FA treatment, did not show any significant changes following CE administration. This could be due to the anti-inflammatory activity of CA and polyphenols, such as catechin family members, in the extract. Western blot analysis also showed that treatment with CE at 200 mg/kg resulted in the elimination of hippocampal phospho-tau and caspase-8. These findings suggest that cinnamon suppresses the FA-induced neuroinflammation, tau hyperphosphorylation, and finally, apoptosis in the hippocampus. 

In a study by Peterson et al*.* (2009[39]), the aqueous extract of CZ inhibited the aggregation of tau protein in the microtubule-binding domain and C-terminal tail of human tau *in vitro*. Moreover, their results showed that the CZ extract contributed to the disassembly of previously formed tau filaments, derived from the brain of AD patients. Although this study was performed *in vitro,* unlike our *in vivo* study, and the proposed stages of tau aggregation and the underlying mechanism were essentially different in these two studies, the final results were consistent. Researchers have found that cinnamon acts as an anti-inflammatory and antioxidant agent in the brain tissue (Ho et al., 2013[16]). Also, it has been shown that CA actively interacts with tau protein and improves the dissociation of NFTs (George et al., 2013[14]). 

By decreasing neuroinflammation and tau hyperphosphorylaton, cinnamon positively affects the extrinsic pathway of apoptosis triggered by FA. This results in the preservation of pyramidal neuron death in the hippocampus, which can explain the improvement of MWM outcomes. Promising results were exclusively observed in the group treated with 200 mg/kg of CE. Researchers strongly believe that CE benefits FA-induced neurotoxicity through a U-turn mechanism. It has been shown that lower doses of CE (100 mg/kg) do not provide adequate bioactive compounds to induce the desired effect, and higher doses (400 mg/kg) cause toxicity. Also, the methanolic extract, unlike the aqueous and essential oil, which mostly consist of either hydrophilic or hydrophobic molecules, contains both types of these molecules and is more likely to contain unwanted compounds with toxic potentials.

We found that the orally administered cinnamon extract improved cognitive performance in FA-treated rats by regulating a series of cellular and molecular events, especially tau protein phosphorylation. It has been shown that the severity of dementia is correlated with the accumulation of NFTs in the brain, mostly phospho-tau (Kolarova et al., 2012[22]). One of the most documented pathways, crucial for triggering tau protein phosphorylation, is the PI3K/AKT/GSK-3β pathway. GSK-3β is an important kinase, which phosphorylates tau protein in response to stress. Moreover, phosphorylated AKT activates GSK-3β, and in turn, the activated GSK-3β phosphorylates various tau epitopes, such as Thr^181^, Thr^231^, Ser^199^, Ser^396^, Ser^400^, Ser^404^, and Ser^413^ both *in vitro* and *in vivo* (Kolarova et al., 2012[22]). 

Thr^231^, the epitope evaluated in the present study, is one of the most important epitopes because of conformational protein changes after phosphorylation, which allow GSK-3β or other kinases to further phosphorylate tau proteins (Kolarova et al., 2012[22]); therefore, elimination of phosphorylation may give us the opportunity to decelerate the progression of disease. Since researchers have proposed the contribution of dietary factors to the regulation of PI3K/AKT/GSK-3β pathway (Calcul et al., 2012[9]), we believe that it might be one of the most potentially involved pathways. However, further investigations are required to reveal the exact mechanism(s) underlying the reduction of tau hyperphosphorylation. It is worth mentioning that despite a slight improvement, the CZ extract had no significant impact on the disturbed memory consolidation, assessed by the inhibitory avoidance test; this finding may highlight some differences in the involved mechanisms.

## Conclusion

This study indicated that the cinnamon extract had favorable effects on neuroinflammation and tau protein hyperphosphorylation. Also, it exerted inhibitory effects on the extrinsic pathway of apoptosis. Overall, cinnamon can be potentially used as a dietary supplement for AD patients; however, further assessments are essential.

## Acknowledgements

This research was fully supported by the Research and Technology Deputy of Guilan University of Medical Sciences.

## Conflict of interest

The authors declare no conflict of interest.

## Authors’ contributions

EN, AZ, and SS contributed to the study design, data collection/analysis, and drafting of the manuscript. PB contributed to behavioral assessments. FY and NA contributed to plant extraction and phytochemical analyses. All authors contributed to the finalization of the manuscript and approved the final version.
